# Testing an Intervention to Improve Posthospital Outcomes in Persons Living With Dementia and Their Family Care Partners

**DOI:** 10.1093/geroni/igad083

**Published:** 2023-08-16

**Authors:** Marie Boltz, Jacqueline Mogle, Ashley Kuzmik, Rhonda BeLue, Douglas Leslie, James E Galvin, Barbara Resnick

**Affiliations:** Ross and Carol Nese College of Nursing, The Pennsylvania State University, State College, Pennsylvania, USA; College of Behavioral, Social, and Health Sciences, Clemson University, Clemson, South Carolina, USA; Ross and Carol Nese College of Nursing, The Pennsylvania State University, State College, Pennsylvania, USA; College for Health, Community, and Policy, The University of Texas at San Antonio, San Antonio, Texas, USA; Center for Applied Studies in Health Economics, The Penn State College of Medicine, State College, Pennsylvania, USA; Miller School of Medicine, Comprehensive Center for Brain Health, University of Miami, Boca Raton, Florida, USA; School of Nursing, University of Maryland, Baltimore, Maryland, USA

**Keywords:** Acute care, ADRD, Dyads, Family engagement, Functional recovery, Transitional care

## Abstract

**Background and Objectives:**

Hospitalized persons living with dementia are at risk for functional decline, behavioral symptoms of distress, and delirium, all persisting in the postacute period. In turn, family care partners (FCPs) experience increased anxiety and lack of preparedness for caregiving, compounding existing strain and burden. Family-centered Function-focused Care (Fam-FFC) purposefully engages FCPs in assessment, decision-making, care delivery, and evaluation of function-focused care during and after hospitalization (within 48 hours of discharge, weekly telephone calls for a total of 7 additional weeks, then monthly for 4 months). The objective of this study was to test the efficacy of Fam-FFC.

**Research Design and Methods:**

A cluster randomized controlled trial included 455 dyads of persons living with dementia and FCPs in 6 medical units in 3 hospitals. Patient outcomes included return to baseline physical function, behavioral symptoms of distress, depressive symptoms, and delirium severity. Family care partner measures included preparedness for caregiving, anxiety, strain, and burden.

**Results:**

Multilevel level modeling demonstrated that the likelihood of returning to baseline function across time for Fam-FFC participants was twice that of the control group by the end of 6 months (OR = 2.4, *p* = .01, 95% CI 1.2–4.7). Family-centered Function-focused Care was also associated with fewer symptoms of distress (*b* = −1.1, *SE* = 0.56, *p* = .05) but no differences in the amount of moderate physical activity, depressive symptoms, and delirium severity. Preparedness for caregiving increased significantly only from 2 to 6 months (*b* = 0.89, *SE* = 0.45, *d* = 0.21, overall *p* = .02) in the intervention group, with no group differences in anxiety, strain, and burden.

**Discussion and Implications:**

Family-centered Function-focused Care may help prevent some of the postacute functional decline and behavioral symptoms in hospitalized persons living with dementia. Further research is needed to promote sustained improvements in these symptoms with more attention to the postacute needs of the care partner.

**Translational Significance:** Hospitalization in persons living with dementia is associated with functional decline, with increased care dependency, and family care partner (FCP) stress during the hospitalization and postacute period. Findings of this study suggest that the Family-centered Function-focused Care (Fam-FFC) intervention, which engages the FCP in function-focused goal setting, care planning, and evaluation supports return to baseline function and less behavioral symptoms of distress, with trends toward increased preparedness for caregiving. However, Fam-FFC did not show improvements in FCP anxiety, strain, or burden. Future refinements of the intervention need to include adaptations to Fam-FFC that strengthen FCP well-being with implementation strategies that promote scalability.

## Background and Objectives

Persons living with dementia are twice as likely to be hospitalized as persons without dementia and comprise one-fourth of hospitalized older adults (1,2). Once hospitalized, individuals living with dementia are more likely to experience functional decline, delirium, and behavioral symptoms of distress (3), all associated with increased rates of rehospitalizations, higher costs, increased morbidity, and earlier mortality (3,4). During the postacute period, persons living with dementia are likely to continue to experience hospital-acquired complications including functional decline and delirium, with increased care needs, and lower quality of life for both them and their family care partner (FCP) (4–8). Prior to hospitalization, approximately 75% of hospitalized patients living with dementia are living at home and receiving care from family members or friends (1,9). The additive stress of hospitalization, and the patient’s increased care dependency, compounds FCP anxiety, strain, and burden (5,6,10).

In hospital settings, acute illness superimposed upon baseline cognitive and functional vulnerabilities accounts for only some of this physical and cognitive loss. Other factors associated with negative outcomes include: (i) inadequate assessment of patient and FCP needs and preferences (including those related to mobility, comfort, and sensory support) (11); (ii) staff underprepared to care for persons with cognitive impairment (11–15); (iii) activity-restrictive policies and environments (12,13,15,16); and (iv) patient, staff, and FCP attitudes regarding physical activity in the hospital setting (11,13–16).

Function-focused care is a philosophy of care wherein staff are taught to care for patients in a way that engages them in performing physical activity and functional tasks at their highest levels (17). Family-centered Function-focused Care (Fam-FFC) builds on this approach by incorporating the family, who can play a critical role in promoting comfort, sense of well-being, and optimizing function of the patient. Previous work showed that during hospitalization FCPs provided critical information about baseline function and cognition, advocated for the patient, and provided encouragement and motivation for the person to be mobile and to engage in physical activity during and after the hospital stay (18). Family-centered Function-focused Care adapts function-focused care to the specialized needs of hospitalized persons living with dementia and also provides transitional care and postacute follow-up that focuses on FCP preparedness to optimize postacute recovery of the patient as well as general caregiving.

The purpose of this study was to test the efficacy of Fam-FCC based on the following hypotheses: (i) Patients who participated in Fam-FFC would demonstrate return to baseline function (i.e., performance of activities of daily living 2 weeks prior to admission), more physical activity, and less delirium, depressive symptoms, and behavioral symptoms of distress, as compared to patients hospitalized on units receiving Fam-FFC education only; and (ii) FCPs who participated in Fam-FFC would demonstrate more preparedness for caregiving, and less anxiety, strain, and burden, as compared to FCPs who did not participate in Fam-FFC.

## Intervention

Family-centered Function-focused Care was coordinated and implemented by a Fam-FFC Nurse, who worked on the treatment units 30 hours a week (7 days a week) for 12 months to implement the 4 components of the intervention, as described in Table 1. Two nurse champions, representing both day and evening shifts on each unit were identified to work with the Fam-FFC Nurse to implement and ultimately sustain the intervention. The 4 components of Fam-FFC include: (I) Environment and policy assessment (to promote function and family engagement); (II) Education of staff; (III) Ongoing mentoring and motivation of staff, patients, and family; and (IV) Completion of Fam-Path Care pathway. The Fam-FFC nurse worked with the FCPs and patients daily during the hospitalization. The number of postacute follow-ups of the nurse interventionists ranged from 7 to 12, with an average of 9.2 contacts per FCP.

**Table 1.** Description of the Family-Centered Function-Focused Care (Fam-FFC) Intervention

Component	When Delivered/by Who	Description
I. Environmental and Policy Assessment	Beginning of the study (the first month of study) and completion of implementation By the Fam-FFC Research Nurse with champions	*Modifications* included development of policies for: labeling glasses/hearing aids, uninterrupted quiet times, family care partner (FCP) involvement in rounds, and bedside white boards to promote FCP/patient communication with the interdisciplinary team; and access to hearing amplifiers, magnifiers, activity cart/supplies; mobility devices and seating; noise trackers; snacks and fluids. Ensuring adequate lighting and clear pathways for walking
II. Staff Education and Training (delivery options include: instructor-led PowerPoint presentations, web-based training, and one-on-one review)	Beginning of the study (during the first 2 mo of study) By the Fam-FFC Research Nurse on intervention units; alternate nurse on control units	*Content* included: Dementia, delirium, functional decline etiology, cognitive/functional assessment, communication, and evidence-based approaches to prevent iatrogenesis and behavioral symptoms of distress Hospital experience and responses of the person with dementia and family Function-focused care (FFC) (rationale, FFC int routine, specific techniques/equipment, safety considerations, goal setting/discharge planning) Partnerships with families (assessment of preferences, listening, information-sharing, care planning, promoting advocacy and engagement in decision-making, discharge planning)
IV. Ongoing Training and Motivation of Nursing Staff	Following initial education of the staff; during 12 mo of implementation By Fam-FFC Research Nurse mentors the unit champions and nursing staff	Components include: *Assistance to champions and nurses* is provided on consented patients to: (a) assess physical capability and cognition; (b) establish and update FFC goals with input from FCPs/patients (Goal Attainment scale); and (c) develop a care plan with FCP/patient addressing factors that impede FFC (eg, sedation, pain, fear/anxiety, pain, apathy) *Support of the unit champions to mentor nursing staff* (RN, LPN, nursing assistants) includes: (a) role modeling; (b) highlighting staff role models; (c) garnering support by sharing success stories with nursing council and administration; (d) educational boosters via brief engaging emails and maintaining Fam-FFC bulletin board with updates/educational reinforcement; and (e) observing/providing feedback to nursing staff during care interactions using the Function-Focused Care Behavior Checklist
IV. Fam-Path Care Pathway	During the 12 mo of implementation By the Fam-FFC Research Nurse	Components of Fam-Path include: *Information* on the admitting condition, diagnostics, and treatment (specific to patient’s unique situation) *Family/patient education (standardized)*: impact of hospitalization upon the person living with dementia, support of physical activity and cognitive stimulation, cueing and motivating, nutritional and sleep promotion, behavioral support, safety, linked to joint FCP/nurse assessment (baseline cognitive/ physical function); discharge teaching *Jointly developed* bedside goals and treatment plans (updated daily) based upon preferences of patient/FCP *Coaching of primary nurse* to communicate to postacute providers *Postacute follow-up*: ongoing education and modification of the function-focused care plan (within 48 h of discharge, weekly telephone calls for a total of 7 additional weeks, then monthly for 4 mo), coaching, and resources for FCPs, all based upon FCP/patient reported needs and preferences

*Note*: Information/teaching requested by FCPs and provided to support preparedness for caregiving: (i) emotional and behavioral support to care receiver; (ii) physical and cognitive activity (within a structured, daily routine); (iii) the advocacy role; and (iv) access to home resources (including respite care, food/meals, and transportation).

The Attention Control condition (Fam-FFC Ed-only) consisted of education of the nursing staff per manualized protocol in participating hospital units. The education was offered exactly as in treatment sites, provided by research staff not familiar nor engaged with the intervention. Education of FCPs included orientation to the hospital and traditional discharge teaching (medications/treatments, medical follow-up).

## Conceptual Model

The Fam-FFC intervention was developed using a social ecological framework (19,20) that acknowledges the intrapersonal (intrinsic), interpersonal (related to patient, family, and nursing staff interaction), and environmental and policy/process factors that influence function in hospitalized persons living with dementia. Social Cognitive Theory (SCT) is used at the interpersonal level to facilitate behavior change (21). Social Cognitive Theory suggests that the stronger the individuals’ self-efficacy and outcome expectations, the more likely it is that they will initiate and persist with a given activity. Self-efficacy expectations are the individuals’ beliefs in their capabilities to perform an activity to attain a desired outcome; and outcome expectations are the beliefs that certain outcomes will be acquired by their actions. Efficacy expectations are dynamic and enhanced by 4 mechanisms: (i) successful performance of the activity; (ii) verbal persuasion; (iii) seeing like individuals perform a similar activity; and (iv) pleasant physiological and affective states (eg, pain reduction) associated with an activity (21). These 4 mechanisms are incorporated into Fam-FFC to motivate patients to be functionally and cognitively active; education and coaching are provided by the Fam-FFC nurse to both staff and FCPs to utilize these mechanisms.

## Research Design and Methods

### Study Design

This was a randomized clinical trial with hospital units randomized by clustering to either treatment (Fam-FFC) or the control condition (Fam-FFC Ed-only). The protocol has been published (22) and follows the Standard Protocol Items: Recommendations for Interventional Trials (SPIRIT) Guidelines (23). It was approved by the university’s Institutional Review Board and registered with clinicaltrials.gov (NCT03046121). The study was monitored by an independent Data Safety and Monitoring Committee.

### Settings/Sample

Six medical units of 3 hospitals (a large academic medical center, a medium-sized teaching hospital, and a small community hospital) and 461 patients and their FCPs participated in the study. The units were similar in terms of patient population (representation of older adults with dementia and need for general medical care) and nurse staffing (nurse–patient ratio of 1:5 on average). Patient inclusion criteria included age ≥65 years, speaking English or Spanish, live in the community prior to admission to the hospital, screen positive for dementia on well-validated scales (Montreal Cognitive Assessment [MoCA] ≤25 (24,25) and AD8 ≥2) (26), have a diagnosis of very mild-to-moderate/stage dementia (confirmed by the Pfeffer Functional Activities Questionnaire [FAQ]) (27) to discriminate mild cognitive impairment (MCI) from dementia and a score of 0.5 to 2.0 on the Clinical Dementia Rating scale (CDR) (28), and have a designated study partner for the duration of the study. Patients were excluded from the study if they had MCI (CDR = 0.5 without functional or impairments in activities of daily living), severe dementia (CDR = 3), any significant neurological condition associated with cognitive impairment other than dementia (eg, brain tumor), a major acute psychiatric disorder, had no FCP to participate, were enrolled in a hospice, admitted from a nursing home, or transferred to another unit for stays longer than 48 hours.

FCPs were eligible to participate if they were age 18 years and older, spoke English or Spanish, were related to the patient by blood, marriage, adoption, or affinity as a significant other (defined by the legally authorized person as the primary person providing oversight and support on an ongoing basis), were able to recall at least 2 words of a 3-word recall (to eliminate cognitive impairment), and participated, at a minimum, in the initial assessment and development of the plan of care.

### Recruitment

After study information was provided the Evaluation to Sign Consent (29) was completed by the patient and FCP. If decisional capacity was impaired and the patient provided assent, the legally authorized representative completed the consent process for the patient and themselves. Upon consent, the research evaluator checked FCP eligibility and then conducted the dementia screening for the patient. Enrollment and intervention materials were available in Spanish for participants, but not required as all participants were fluent in and preferred English-speaking materials.

**Figure 1.**
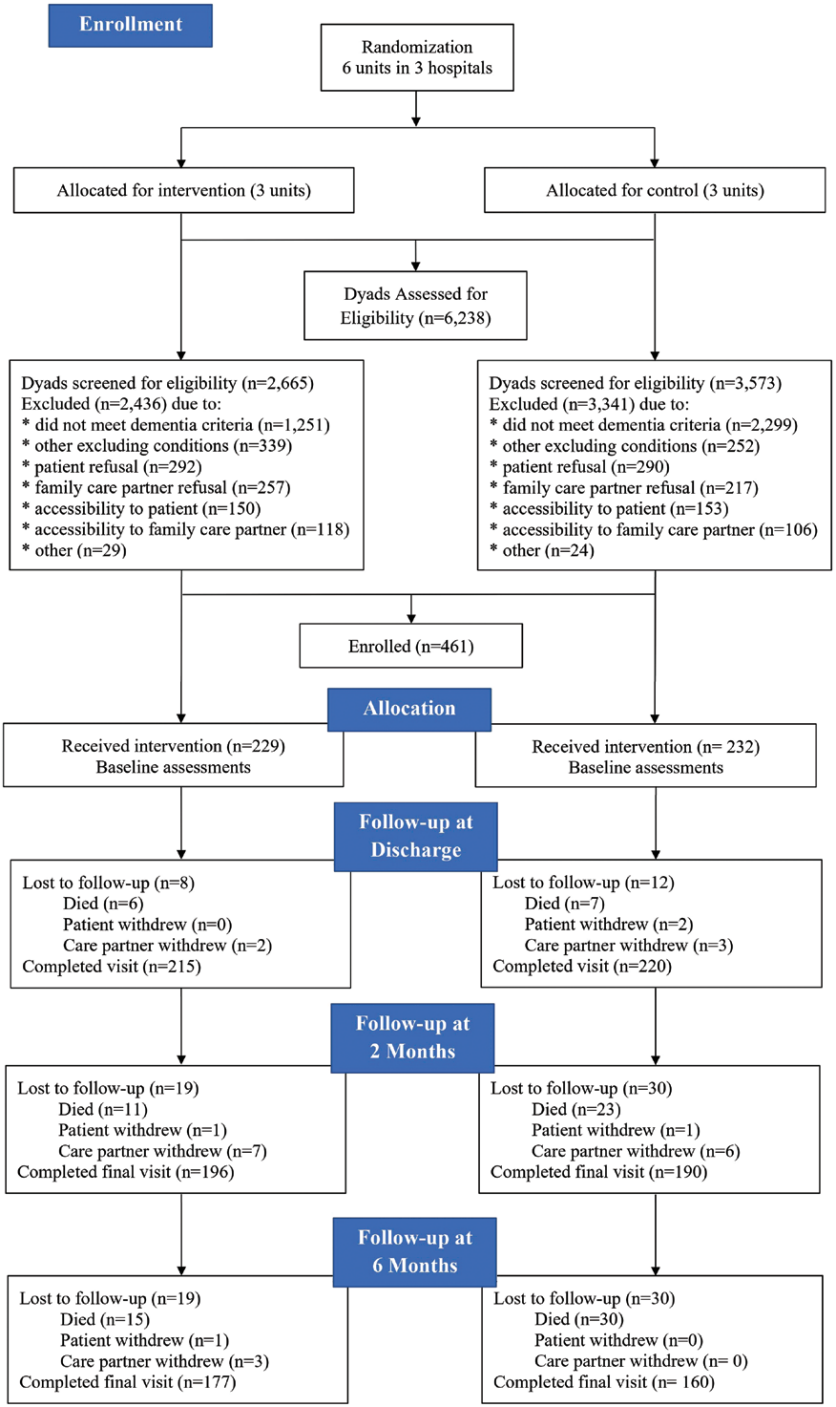
Consolidated Standards of Reporting Trials (CONSORT) flow diagram.

As shown in Figure 1, a total of 6 238 patients, aged 65 and older, were prescreened and 2 261 (36%) were eligible to approach for consent and additional screening. Of the 1 775 that were approached and screened, 608 (34%) dyads consented. A total of 461 dyads (26% of those approached and eligible, and 76% of those consented) enrolled. This analysis did not include 6 participants (3 White Hispanic and 3 Asian) due to their low representation giving a final sample of 455 dyads. The attrition rate was 26% with the majority due to deaths (*n* = 45/48 = 94%).

### Procedures

Participants were blinded to treatment arm prior to consent. Evaluators were not informed of randomization results or provided with the details of the intervention. Enrolled patients were evaluated within 48 hours of admission, 72 hours of discharge, and 2 and 6 months after discharge through a combination of chart audit, information from FCPs, and observational measures. Family care partner data were collected at 72 hours of patient’s discharge, and 2- and 6-months postdischarge via FCP report.

### Measures

#### Descriptive measures

Patient descriptive measures include age, race, ethnicity, gender, marital status, educational level, disease burden (Charlson Comorbidity Index) (30), cognitive status assessed with the MoCA, and length of stay (time from admission to discharge, as a difference).

Preadmission function (ADL performance 2 weeks prior to admission) using the Barthel Index (31) was evaluated based on a report from the FCP. Family care partner descriptive measures included age, race/ethnicity, gender, marital status, education, employment status, kinship to patient, all acquired at the time of the first visit. General health and change in health compared to 1 year ago are assessed using individual items from the SF-36 (32).

#### Patient outcome measures

Outcome measures were evaluated within 48 hours of admission, within 72 hours of discharge, and 2 and 6 months after discharge, and included return to baseline physical function (the primary outcome), physical activity, delirium, mood, behavioral symptoms, and hospital admissions. Physical function was assessed using the Barthel Index (31) based on verbal report from the FCP. Return to baseline function was based on return to the Barthel Index report related to 2 weeks prior to admission or improvement and coded as return to preadmission functioning (1) or not (0). Physical activity was measured for 24 hours using the MotionWatch8. The MotionWatch8 is a wrist-worn actigraphy monitor that records activity in set epochs of time with established reliability and validity and is well tolerated by cognitively impaired older adults (33). Activity is recorded as sedentary, low activity, and moderate activity using the Landry cutpoints. We evaluated counts of moderate activity (562 counts/min), which include activities ≥3 metabolic equivalents, such as walking at 100 steps/minute, because moderate activity has been shown to be associated with return to baseline function (33).

Delirium severity was measured with an additive score for the 4 items of the Confusion Assessment Method Severity (CAM-S) Short Form (34). Mood was assessed using the 19-item Cornell Scale for Depression in Dementia (CSDD) (35). Behavioral symptoms were evaluated with the 12-item Brief Neuropsychiatric Inventory (NPI-Q) Severity scale, informed by FCPs (36).

Although not primary outcomes, we also examined the number of falls, hospitalizations (admissions to the hospital), and emergency department (ED) visits (ED-only), acquired from the report of the FCP at each postacute data collection point.

#### Family care partner outcomes

Family care partner outcomes were preparedness for caregiving (the primary outcome), anxiety, strain, and burden. The Preparedness for Caregiving scale is an 8-item instrument that asks FCPs how well prepared they believe they are for multiple domains of caregiving: physical care and emotional support, setting up support services, dealing with the stress of caregiving. Items are rated 0 (not at all prepared) to 4 (very well prepared) (37). Anxiety was evaluated with the 7-item Hospital Anxiety and Depression scale (HADS) subscale for Anxiety (HADS-A) (38). Strain was assessed with the Modified Caregiver Strain Index, a 13-question tool that measures strain (financial, physical, social, psychological, and personal) related to care provision (39). Family care partner burden was measured with the Zarit Burden Interview (ZBI-12) short version, a 12-item tool that measures perception of burden has shown high internal consistency and discriminative ability in persons caring for a person living with dementia (40). All measures show acceptable psychometric properties in the study population and are reported in the published protocol (22).

We also conducted a conventional content analysis (41) of the nurse interventionist daily documentation to examine the nature of the support provided by the interventionists.

### Data Analysis

Intention-to-treat analysis was used. If the participant died or withdrew from the study, already-collected data were not removed from the study database. For patients who died, data obtained up until the time of death were included in the analyses. Power analysis was based on simulated multilevel models to appropriately account for the dependency among observations (SAS 9.4). Simulations indicated that a sample of 438 was sufficient to detect group differences in slopes (ie, change across time due to intervention) as small as 0.182 in the patient group with 4 timepoints and as small as 0.2 in the FCP group with 3 timepoints.

Prior to analysis, variables were screened for outliers and descriptive statistics were computed across both groups. Consistent with best practices, we also examined intraclass correlations and examined multilevel growth models prior to primary analyses.

Primary multilevel models included intervention (1) vs control (0) group as a main effect to test for differences among groups at baseline. An interaction between intervention and time because baseline identified whether there were group differences in change across time. Within each model, we also computed specific a priori contrasts comparing groups at each time point to provide evidence of efficacy. Patient behavioral symptoms and all FCP outcomes were treated continuously. Return to baseline function was scored as return to or improvement in preadmission (2 weeks prior) functioning (1) or not (0) (using the Barthel) (31,42) and was modeled using a binomial distribution with a logit link. Delirium severity, falls, ED visits, and hospitalizations were treated as count variables and were modeled using a Poisson distribution with a log link. For effect sizes continuous outcomes are represented as standardized differences; binomial and Poisson outcomes are odds and rate ratios, respectively. Patient models included age, gender, race, comorbidity, cognitive status, and dementia severity as covariates. Family care partner models included FCP age, gender, and race, as well as patient cognitive status, dementia severity, and preadmission functioning as covariates in the model. Age, comorbidities, and cognitive status were grand mean-centered and categorical covariates were effect coded for interpretation.

## Results

### Participant Characteristics

Dyad characteristics are reported in Table 2. The majority of patients identified as female, with an average age of 81.5 (±8.4); approximately 64% were White patients and 36% were Black or African American patients. The sample indicated, on average, moderate impairment in cognition (MMoCA = 11.7 ± 7.0) and baseline (preadmission) some impairment in physical function (MBI = 78.1 ± 22.6), mild pain (MPAINAD = 0.79 ± 1.5), and depression (MCESD = 9.1 ± 6.2). There was significantly more comorbidity in the control group (MCCI = 4.6 ± 2.9) as compared to the intervention arm (MCCI = 4.1 ± 2.6) and more severe behavioral symptoms at baseline in the intervention group (MNPI-Q = 8.4 ± 6.6), as compared to the control group (MNPI-Q = 7.2 ± 5.5). Approximately one-third of the patients in both groups demonstrated moderate or severe delirium.

**Table 2.** Characteristics of the Dyads

Variable	Total	Intervention (*n* = 223)	Control (*n* = 232)	*p* Value
*Patients/persons with dementia (N = 455)*				
Female, *n* (%)	269 (59.1)	139 (62.3)	130 (56.0)	.22
Race, *n* (%)				.12
White	290 (63.7)	134 (60.0)	156 (67.2)	
Black/African American	165 (36.3)	89 (40.0)	76 (32.8)	
Marital status, *n* (%)				.48
Widowed	191 (42.0)	93 (41.7)	98 (42.2)	
Married	169 (37.1)	81 (36.3)	88 (37.9)	
Other	95 (20.9)	49 (22.0)	46 (19.9)	
Education, *n* (%)				.23
Less than high school	90 (19.7)	43 (19.4)	47 (20.3)	
High school, trade school, some college	252 (55.3)	134 (60.0)	118 (50.8)	
College and beyond	101 (22.2)	40 (17.9)	61 (26.3)	
Chose to not answer	12 (2.7)	6 (2.7)	6 (2.6)	
Age, mean (*SD*)	81.5 (8.4)	82 (8.4)	81.1 (8.3)	.26
Length of hospital stay, mean (*SD*)	6.5 (4.4)	6.6 (4.6)	6.5 (4.1)	.94
*Family care partners (N = 455)*				
Female, *n* (%)	329 (72.3)	161 (72.2)	168 (72.4)	.95
Race, *n* (%)				.26
White	291 (64.0)	134 (60.0)	157 (67.7)	
Black/African American	153 (33.6)	84 (37.7)	69 (29.7)	
More than one	5 (1.1)	3 (1.3)	2 (0.9)	
Not reported or unknown	6 (1.3)	2 (1.0)	4 (1.7)	
Marital status, *n* (%)				
Married	268 (58.9)	129 (57.8)	139 (59.9)	
Education, *n* (%)				.44
Less than high school	34 (7.5)	14(6.3)	20 (8.6)	
High school, trade school, some college	237 (52.1)	124 (55.6)	113 (48.7)	
College and beyond	162 (35.6)	75 (33.6)	87 (37.5)	
Chose to not answer	22 (4.8)	10(4.5)	12 (5.2)	
Kinship with patient, *n* (%)				.39
Spouse	130 (28.6)	63 (28.3)	67 (28.9)	
Daughter	169 (37.1)	77 (34.5)	92 (39.7)	
Son	67(14.7)	37 (16.6)	30 (12.9)	
Other	89(19.6)	46 (20.6)	43 (18.5)	
Lives with patient, *n* (%)	270 (59.3)	136 (60.0)	134 (57.8)	.51
Works outside the home, *n* (%)	190 (41.8)	100 (44.8)	90 (38.8)	.21
Age, mean (*SD*)	61.8 (14.2)	61.1 (14.2)	62.6 (14.1)	.28
Perception of health, mean (*SD*)	3.1 (0.9)	3.1 (0.9)	3.1 (0.9)	.86
Change in health as compared to one year ago, mean (*SD*)	3.0 (0.8)	3.0 (0.8)	2.9 (0.7)	.10

*Note*: *SD* = standard deviation.

The majority of FCPs identified as female, lived with the patient, and were spouses or daughters. Approximately two-thirds identified as White adults and one-third as Black or African American adults. Their average age was 61.9 (±14.2) and they described their general health as good (MSF-36 = 3.1 ± 0.90) and unchanged from a year ago (MSF-36 = 3.0 ± 0.77).

### Outcomes

Tables 3–5 report all a priori-specific contrasts from the multilevel models for patient and FCP outcomes.

**Table 3.** Outcome Measures by Patient Outcomes

Variable	Control		Intervention		Odds ratio (95% CI)	Differences	*p* Value
	Odds (*SE*)	Mean (*SE*)	Odds (*SE*)	Mean (*SE*)			
Return to baseline function at 6 mo							
Admission	0.65 (0.09)		0.57 (0.09)				.22
Discharge	0.62 (0.09)		0.68 (0.08)				.38
2 mo	0.49 (0.09)		0.67 (0.09)				.01
6 mo	0.41 (0.91)		0.54 (0.10)				.07
Return to baseline function (intervention vs control)					2.39 (1.22–4.68)		.01
Physical activity (moderate activity minutes)							
Admission		8.4 (6.1)		4.6 (6.2)		3.0 (4.1)	.35
Discharge		17.4 (6.4)		14.6 (6.5)		2.8 (4.8)	.56
2 mo		27.8 (6.8)		22.9 (6.6)		4.9 (5.4)	.37
6 mo		23.3 (7.2)		25.1 (6.7)		−1.9 (6.1)	.76
Change admission to 6 mo in intervention vs control						5.8 (6.4)	.37
Delirium							
Admission		1.3 (0.10)		1.6 (0.11)		−0.27 (0.15)	.07
Discharge		1.0 (0.11)		1.0 (0.11)		0.00 (0.15)	.99
2 mo		1.1 (0.11)		1.1 (0.11)		0.05 (0.16)	.73
6 mo		1.0 (0.12)		1.2 (0.12)		−0.12 (0.17)	.47
Change admission to 6 mo in intervention vs control						−0.15 (0.18)	.40
Depression							
Admission		7.1 (0.84)		7.2 (0.86)		−0.06 (0.57)	.92
Discharge		5.7 (0.84)		5.3 (0.86)		0.38 (0.57)	.50
2 mo		5.13 (0.85)		4.1 (0.86)		0.99 (0.60)	.10
6 mo		3.84 (0.86)		4.2 (0.87)		−0.39 (0.63)	.54
Change admission to 6 mo in intervention vs control						−0.33 (0.64)	.61
Behavior							
Admission		4.2 (0.82)		3.8 (0.83)		0.40 (0.55)	.47
Discharge		3.5 (0.83)		2.8 (0.84)		0.72 (0.58)	.21
2 mo		3.0 (0.84)		3.1 (0.84)		−0.14 (0.60)	.82
6 mo		5.1 (0.82)		5.8 (0.83)		−0.72 (0.54)	.19
Change admission to 6 mo in intervention vs control						−1.1 (0.56)	.05

*Notes*: CI = confidence interval; *SE* = standard error.

**Table 4.** Outcome Measures: Adverse Events by Intervention Arm

Variable	Estimate	*SE*	*z* Value	*p* > IzI	Exponentiated
Total ER visits					
Control	−0.36	0.20	−1.84	0.07	0.69
Intervention	0.23	0.22	−1.06	0.29	0.80
Differences	−0.15	0.09	−1.70	0.09	0.87
Total falls					
Control	0.22	0.24	−0.94	0.35	0.80
Intervention	0.12	0.19	0.63	0.53	1.13
Differences	−0.34	0.42	−0.82	0.41	0.71
Total hospitalizations					
Control	−0.39	0.24	1.64	0.10	0.67
Intervention	−0.49	0.24	−2.09	0.04	0.61
Differences	0.01	0.06	1.85	0.06	1.11

*Notes*: ER = emergency room; *p* = *p* values; IzI = z score; *SE* = standard error.

**Table 5.** Outcome Measures: Family Outcomes

Variable	Control	Intervention	Differences	*p* Value
	Mean (*SE*)	Mean (*SE*)		
Prepared				
Discharge	24.3 (1.8)	24.2 (1.8)	0.09 (0.64)	.89
2 mo	25.1 (1.8)	24.6 (1.8)	0.48 (0.67)	.47
6 mo	26.0 (1.8)	27.0 (1.8)	−1.00 (0.69)	.15
Change discharge—6 mo: intervention vs control			−1.09 (0.63)	.08
Change 2–6 mo: intervention vs control			−1.48 (0.62)	.02
Anxiety				
Discharge	3.0 (1.2)	2.9 (1.2)	0.11 (0.45)	.80
2 mo	2.5 (1.2)	2.4 (1.2)	0.11 (0.47)	.81
6 mo	2.6 (1.2)	2.7 (1.2)	−0.07 (0.50)	.90
Change discharge—6 mo: intervention vs control			−0.02 (0.45)	.70
Strain				
Discharge	3.1 (1.7)	2.7 (1.8)	0.45 (0.63)	.48
2 mo	2.6 (1.8)	2.7 (1.8)	−0.06 (.650)	.92
6 mo	2.4 (1.8)	1.7 (1.8)	0.70 (0.67)	.30
Change discharge—6 mo: intervention vs control			0.25 (0.58)	.66
Burden				
Discharge	4.9 (2.5)	4.6 (2.5)	0.45 (0.62)	.48
2 mo	8.4 (2.5)	8.4 (2.5)	−0.06 (0.65)	.92
6 mo	3.9 (2.5)	4.0 (2.6)	0.70 (0.67)	.30
Change discharge—6 mo: intervention vs control			0.25 (0.58)	.66

*Note*: *SE* = standard error.

#### Patient outcomes

*Functioning*.—The odds of returning to baseline function significantly decreased across the study period from 0.61 (*SE* = 0.08) at admission to 0.47 (*SE* = 0.09) at 6 months (*F*(3,1338) = 6.8, *p* < .01) and this was qualified by an interaction with the intervention condition (*p* = .009). The intervention group was 2 times more likely to have returned to baseline function at 2 months (OR = 2.1, *p* < .01, 95% CI 1.2–3.7). A comparison of return to baseline function across time for intervention and control groups indicated intervention participants were more than twice as likely to return to baseline function by the end of 6 months (OR = 2.4, *p* = .01).

Moderate physical activity significantly increased across the study period from 5.6 (*SE* = 2.1) at admission to 23.3 (*SE* = 3.1) at the 6-month follow-up (*F*(3,529) = 18.1, *p* < .001, *d* = 0.59), but this increase did not differ across intervention and control (*p* = .78). Delirium severity significantly decreased across the study period from 0.70 (*SE* = 0.12) at admission to 0.54 (*SE* = 0.09) at 6 months (*F*(3,1131) = 14.3, *p* < .01) and was similar across groups (*p* = .22).

*Distress.—*Behavioral symptoms of distress significantly decreased across the study period from 7.8 (*SE* = 0.28) to 5.4 (*SE* = 0.31) at the 6-month follow-up (*F*(3,1137) = 28.8, *p* < .001, *d* = 0.57). Differences between the arms were apparent when comparing admission levels to 6-month levels (changeintervention = −1.97, *SE* = 0.40, *p* < .001, *d* = 0.48; changecontrol = −0.86, *SE* = 0.39, *p* = .03, *d* = 0.20) and the specific contrast was significant (*b* = −1.1, *SE* = 0.56, *p* = .05) indicating Fam-FFC was associated with fewer behavioral symptoms of distress at 6 months. Depressive symptoms significantly decreased across the study period from 9.1 (*SE* = 0.29) to 6.0 (*SE* = 0.32) at the 6-month follow-up (*F*(3,1137) = 37.3, *p* < .01, *d* = 0.69) and were similar across groups (*p* = .16).

*Adverse events*.—There was no significant difference in the total number of falls or transfers to the ED between the groups. Regarding the total number of hospitalizations across the follow-up period, the control group was approximately 10% more likely (*p* < .06) to have a hospitalization.

#### Care partner outcomes

All FCP outcomes significantly changed from discharge to 6 months (all *p* < .05). Interactions for anxiety, strain, and burden indicated similar change across groups over the study period (all *p* < .41) with no significant treatment effect. Preparedness for caregiving significantly increased from 23.9 (*SE* = 0.32) at discharge to 26.1 (*SE* = 0.34) at 6 months (*d* = 0.53). Preparedness for caregiving increased significantly more from the 2-month to the 6-month assessment (changeintervention = 2.4, *SE* = 0.44, *d* = 0.56; changecontrol = 0.89, *SE* = 0.45, *d* = 0.21, overall *p* = .02) in the Fam-FFC arm, and increased from discharge to 6 months (changeintervention = 2.8, *SE* = 0.44, *d* = 0.68; changecontrol = 1.7, *SE* = 0.44, *d* = 0.42, overall *p* = .08), albeit not significant, in the intervention group.

### Description of Interventionist Activity

Five themes describe the teaching and coaching provided in response to FCP requests and needs. First, family caregivers asked for information on providing emotional and behavioral support. The nurse interventionists addressed 2 areas in their teaching, including helping the care recipient stay socially connected (with family, friends, and participation in adult day care) as well as managing behavioral symptoms of distress. In addition to communication and environmental approaches, the nurses addressed the related second category of needs—helping their family member be physically and cognitively active. The notes describe communication approaches as well a structured, daily routine, and engagement in physical activity (eg, “helping activities,” walking, dancing).

Family care partners also asked for help in managing symptoms, especially detecting delirium (“new confusion”) and promoting good sleep. Another category was supporting the FCPs to act as advocates, especially in the hospital setting. This included “finding the words” to advocate for the care recipient to be out of bed and assisted to walk. After discharge, some FCPs described the need to be more engaged in decisions about medications, including avoiding drugs that caused negative reactions in the past. Finally, the FCPs wanted more home resources to provide respite and help the care receiver be less dependent on their time and attention (43).

### Treatment Fidelity

Treatment fidelity was evaluated in terms of delivery, receipt, and enactment of the intervention (44). Delivery of all 4 components was provided as intended (see Table 1). Receipt was based on Fam-FFC knowledge test scores of greater than 80% among all nursing staff and goal achievement as the majority (74%) of patient goals were achieved as expected or higher than expected (45), and improvement in environments to better support FAM-FFC. Lastly, enactment was based on evidence that in 67% of the observations, participants performed at least one function-promoting behavior.

## Discussion

This study demonstrated preliminary, modest support for the efficacy of Fam-FFC for some of the hypothesized outcomes of hospitalized persons with dementia and their FCPs. Relevant to the primary target of the intervention, patients exposed to Fam-FFC were more likely to return to baseline function over time when compared to those exposed to routine care. This outcome corroborates other work that engaged families in function-focused care during hospitalization and the postacute period and underscores the contribution that FCPs may make to the functional recovery of the person with dementia (17,18). These results are consistent with the goals set by FCPs in this study, which focused on mobility and self-care of the patient and may have contributed to improved outcomes in physical function (42). Results also indicate, however, the need for increased attention from the 2-month period onward to promote sustaining functional gains.

The return to baseline function in the intervention group was not accompanied by an increase in time spent in moderate activity. It may be that the MotionWatch8 was not worn for enough time to capture all the physical activity that occurred during the hospital stay or the physical activity simply did not rise to sufficient levels of activity to meet U.S. Department of Health and Human Services definitions of moderate level physical activity (46). The findings of overall low levels of physical activity are consistent with prior research (33,47,48). Ongoing research is needed to continue to encourage and engage older patients living with dementia in higher levels of activity when hospitalized.

The participants who received Fam-FFC demonstrated fewer behavioral symptoms of distress as compared to the control group at 6 months particularly. Research in long-term care indicated that function-focused care adapted to the needs and preferences of the person living with dementia improved both cognitive and behavioral outcomes (49). As part of the Fam-Care Pathway, FCPs were helped to provide function-focused care, provided in tandem with a structured daily routine and meaningful activities. Findings suggest that this approach may improve both physical and behavioral function after hospitalization of the person living with dementia. Clinically, interdisciplinary discharge interventions and transitional care interventions should focus on expanding discharge planning for hospitalized persons living with dementia and their FCPs to move beyond a focus on medications and follow-up and include FCP preparedness to support functional recovery and optimal management of behavioral distress.

The intervention was not associated with improvements in mood or delirium. Lack of detection of change in depressive symptoms may be due to the relatively low levels of depressive symptoms such that identification of change was difficult, or simply that the intervention did not impact these symptoms or the presentation of delirium. Additionally, we acknowledge that the intervention did not focus on the use of pharmacological and nonpharmacological strategies to manage depression, and medical interventions to manage delirium.

There were fewer hospital readmissions in the intervention group, consistent with previous studies (17,18). There was no evidence to support that Fam-FFC resulted in an increase in falls during and posthospitalization, which is commonly expressed as a concern of hospital staff, patients, and family members, when mobility and physical activity are encouraged.

The FCPs who participated in Fam-FFC showed trends toward increased preparedness when compared to the control group, with increases from 2 to 6 months and a modest albeit nonsignificant increase from discharge to 6 months. Future investigation should examine the impact of Fam-FFC upon preparedness in areas that matter most to FCPs, including attending to the patient’s emotional/behavioral needs, plan for physical and cognitive activity, their role as patient advocate, and need for practical resources (eg, respite care, food/meals, and transportation) (43).

Research is also warranted that examines Fam-FFC in sub-groups of FCPs, considering their distinct roles, kinships, and intrinsic characteristics such as demographics, health literacy, use of resources, social supports, and competing demands. The intervention was not associated with improvements in FCP anxiety, strain, and burden, suggesting the need for interventions that specifically address these symptoms and implement relevant interventions for the long-term FCP (eg, physical activity for the FCPs, respite care) (50) after the hospitalization of the care recipient. Our intervention was focused on promoting the functional recovery of the person with dementia and providing the FCP with resources to support this goal. Our findings clearly suggest the need to include strategies that support the well-being of the FCP, including stress reduction and self-care. Also, the intervention required face-to-face contact with a nurse, was time intensive for both the nurses and care partners and required multiple scheduling efforts. Thus, when refining the intervention, more efficient modes of delivery including the use of a web-based program will be explored.

## Study Limitations and Implications for Practice and Research

The generalizability of findings from this study is limited given the inclusion of a sample of dyads from only 6 units in 3 hospitals. Furthermore, it is likely that there was a recruitment bias and that only those willing to and interested in participating in a study that would help them prevent and/or recover from hospital-acquired complications and improve FCP preparedness participated. Also, as with any longitudinal study of older adults, there was significant attrition during the 6-month follow-up.

The measures used in the study were deficit-oriented. Research is needed to develop, test, and use strength-based measures for both care partners and persons living with dementia to test Fam-FFC and other dyadic interventions. Such work is underway in both Europe and the United States (51,52), consistent with the research and policy priority to enable people living with dementia and their care partners to “live well.”

Despite limitations, this study supports the use of Fam-FFC to improve functional and behavioral outcomes of hospitalized persons with dementia. Further research is needed to promote sustained improvements in these symptoms with more attention to the postacute needs of the care partner, and measures that examine what matters most to the care partner and patient.

## Conclusion

The findings of this study suggest Fam-FFC was beneficial in some regards to both patients and FCPs and may be implemented without increasing the risk of falls and ER transfers, while possibly decreasing hospitalizations. Future research should focus on refining and testing the intervention to better promote the well-being and preparedness of diverse FCPs in diverse settings, and ensuring outcomes are meaningful and sustained for both FCPs and patients.
